# Natural Antioxidant Activities of Plants in Preventing Cataractogenesis

**DOI:** 10.3390/antiox11071285

**Published:** 2022-06-28

**Authors:** Eva Imelda, Rinaldi Idroes, Khairan Khairan, Rodiah Rahmawaty Lubis, Abdul Hawil Abas, Ade John Nursalim, Mohamad Rafi, Trina Ekawati Tallei

**Affiliations:** 1 Graduate School of Mathematics and Applied Sciences, Universitas Syiah Kuala, Banda Aceh 23111, Indonesia; evaimeldaspmpo@gmail.com; 2 Department of Ophthalmology, General Hospital Dr. Zainoel Abidin, Banda Aceh 23126, Indonesia; 3 Department of Ophthalmology, School of Medicine, Universitas Syiah Kuala, Banda Aceh 23111, Indonesia; 4 Department of Chemistry, Faculty of Mathematics and Natural Sciences, Universitas Syiah Kuala, Banda Aceh 23111, Indonesia; 5 Department of Pharmacy, Faculty of Mathematics and Natural Sciences, Universitas Syiah Kuala, Banda Aceh 23111, Indonesia; khairankhairan@unsyiah.ac.id; 6 Department of Ophthalmology, Faculty of Medicine, Universitas Sumatera Utara, Medan 20222, Indonesia; rahma.lubis@yahoo.com; 7 Department of Biology, Faculty of Mathematics and Natural Sciences, Sam Ratulangi University, Manado 95115, Indonesia; abdulabas102@student.unsrat.ac.id (A.H.A.); trina_tallei@unsrat.ac.id (T.E.T.); 8 Department of Ophthalmology, General Hospital Prof. Dr. R. D. Kandou, Manado 955234, Indonesia; dr.adejn@gmail.com; 9 Department of Chemistry, Faculty of Mathematics and Natural Sciences, IPB University, Bogor 16680, Indonesia; mra@apps.ipb.ac.id

**Keywords:** antioxidant, cataract, reactive oxygen species, plants

## Abstract

A cataract is a condition that causes 17 million people to experience blindness and is the most significant cause of vision loss, around 47.9%. The formation of cataracts is linked to both the production of reactive oxygen species (ROS) and the reduction of endogenous antioxidants. ROS are highly reactive molecules produced by oxygen. Examples of ROS include peroxides, super-oxides, and hydroxyl radicals. ROS are produced in cellular responses to xenobiotics and bacterial invasion and during mitochondrial oxidative metabolism. Excessive ROS can trigger oxidative stress that initiates the progression of eye lens opacities. ROS and other free radicals are highly reactive molecules because their outer orbitals have one or more unpaired electrons and can be neutralized by electron-donating compounds, such as antioxidants. Examples of natural antioxidant compounds are vitamin C, vitamin E, and beta-carotene. Numerous studies have demonstrated that plants contain numerous antioxidant compounds that can be used as cataract preventatives or inhibitors. Natural antioxidant extracts for cataract therapy may be investigated further in light of these findings, which show that consuming a sufficient amount of antioxidant-rich plants is an excellent approach to cataract prevention. Several other natural compounds also prevent cataracts by inhibiting aldose reductase and preventing apoptosis of the eye lens.

## 1. Introduction

A cataract is a condition where the eye’s lens clouds and can lead to progressive loss of vision. Cataracts are often associated with age, where with increasing age, the eye’s lens can turn cloudy due to the oxidative stress process, so that vision becomes blurry [1]. Based on age, cataracts are classified into senile, juvenile, and congenital cataracts [2]. Senile cataracts occur at an advanced age (age-related cataracts), juvenile cataracts are categorized when cataracts arise at a young age, and congenital cataracts are cataracts that occur at birth [3,4].

Senile cataract is one of the leading causes of visual impairment and blindness globally and is the most common form of cataract.

The oxidation process plays a vital role in lens opacities in senile cataract cases. The elderly population will increase, increasing the prevalence and incidence of senile cataract cases. Currently, the incidence of senile cataracts is 3.9% at the age of 55–64 years and increases to 92.6% at the age of 80 years and over [5,6].

The prevalence of cataracts as a cause of vision loss increases every year. The World Health Organization (WHO) claims that cataracts are the leading cause of blindness and visual impairment globally, accounting for around 47.9% of the world’s blind people. It is the cause of reversible blindness in more than 17 million (47.8%) of the 37 million blind people worldwide. Cataracts also account for 30–50% of blindness in African and Asian countries [7].

Antioxidants are one of the compounds reported to be able to inhibit the progression of cataracts. Antioxidants react with radical and non-radical species after oxidative stress to trigger defense mechanisms that protect intracellular and extracellular components [8]. Natural antioxidants are created in living cells in nutrition metabolism and immunological function to maintain an oxidation-reduction equilibrium.

Plants provide most natural antioxidants. Plants, which are plentiful in cereals, spices, and essential oils utilized in meat products for organoleptic purposes, are the most abundant source of antioxidants. Tea water extract has also been used as a source of natural antioxidants because it contains several compounds, such as catechins, tannins, and other flavonoids, with the advantage of not having a strong taste like essential oils [9]. Antioxidants and other phytochemicals are abundant in certain fruits and vegetables. Several minerals and vitamins are natural antioxidants because they act as cofactors for antioxidant enzymes. Various short, multifunctional peptides capable of neutralizing free radicals and preventing pro-oxidative metal ions have also been discovered in nature. Antioxidant peptides are produced as a result of the enzymatic breakdown of proteins [8].

Vitamin E, vitamin C, carotenoids, polyphenols, and phenolic compounds may include coumarins, cinnamic acid derivatives, flavonoids, tocopherols, and multifunctional organic acids. The flavonoid molecules flavonols, flavones, isoflavones, catechins, and chalcones are all antioxidants. There are also chlorogenic acid, caffeic acid, ferulic acid, and other cinnamic acid derivatives. The hydroxyl group (-OH) and the double bond are responsible for this property [10].

Based on the description above, in this article, we examined plants reported to have antioxidant activity and have the potential to prevent cataract progression. The most recent review compiled ethnopharmacological/ethnobotanical data on medicinal plants and plant-based natural products used for cataract treatment around the world [11], and another review [12] focused on natural chemicals with antioxidant capabilities that may be used as a large-scale interventional strategy and are also very inexpensive; now, we take a more comprehensive look. This article also includes the most recent updates of several natural products from plants are helpful in preventing cataractogenesis, a process of cataract formation. Antioxidant-containing natural products could be considered potential anticataract agents for the prevention of cataractogenesis. However, as not all natural antioxidants have anticataract properties, they were also studied in a comprehensive manner either in vitro and in vivo.

## 2. Methods

The approach employed is a systematic literature review (SLR) design, which is a systematic literature review by locating, analyzing, and interpreting all results on a single research subject utilizing Google Scholar, Science Direct, PubMed, and Wiley databases.

### 2.1. What Is a Cataract and What Are Cataract Characteristics?

**Figure 1:**
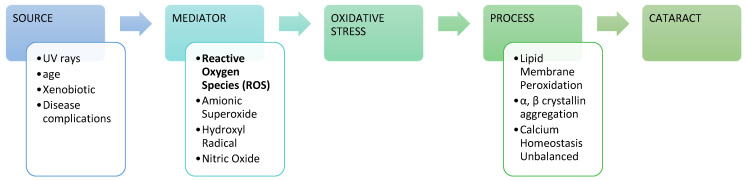
Cataract progression with reactive oxygen species (ROS) mediators [16].

The lens is composed of transparent, flexible tissue and is located directly behind the iris and the pupil. It is the second part of your eye, after the cornea, that helps to focus light and images on your retina. Cataracts are the most common cause of blindness globally, and cataracts are a condition in which lens proteins clump together, causing the lens to become cloudy. Various factors can cause cataracts, but many cases show that free radicals are the mediators behind the pathological processes that lead to cataracts (Figure 1) [13].

Cataracts cause impaired vision function and vision loss because light cannot penetrate the lens. The capsule, lens epithelium, and lens fibers are the three main parts of the lens. Dense connective tissue forms a capsule. The entire lens body is composed of dense, concentric layers of lens fibers. The lens epithelium is a simple cuboidal epithelium that lines the anterior surface of the lens. The lens epithelium plays a crucial role in maintaining homeostasis by allowing ion permeability, nutrients, and osmolarity into the aqueous humor. The primary source of energy for lens tissue is glucose. Lens fiber osmolarity is enhanced by sodium/potassium adenosine triphosphatase and calcium adenosine triphosphatase [14,15].

Numerous factors may produce cataracts. Pathophysiological alterations linked with disorders such as diabetes are a well-known cause of cataract development [17], and some xenobiotics have also been identified as being able to produce cataracts [18]. Cataracts can also be caused by diseases in newborns [19], injury or developmental disorder before birth or during childhood [20], smoking [21], and exposure to harmful substances such as UV rays [22] and corticosteroids [23], among many others.

**Figure 2:**
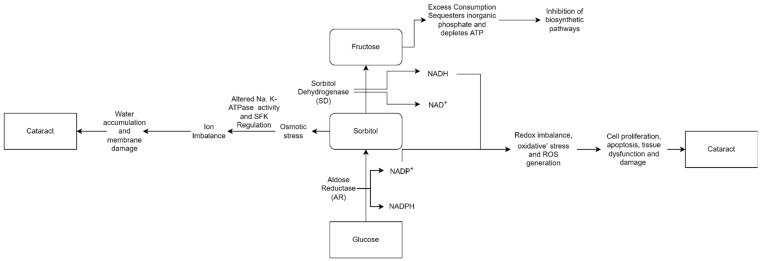
“Osmotic Hypothesis” of sugar cataract formation, relating AR-mediated accumulation of polyols in lens swelling associated with complex biochemical changes, ultimately leading to cataract formation [26].

These various causes allow the development of cataracts to occur by multiple mechanisms as well. Cataracts can occur due to the accumulation of sorbitol. Extracellular glucose diffuses into the lens during hyperglycemia, causing post-translational modifications. Cataract progression is caused by excessive sorbitol synthesis and accumulates in the lens fibers, causing osmotic stress (Figure 2). Sorbitol is produced by aldose reductase using NADPH and cannot cross cell membranes. However, it can accumulate in cells and disrupt the osmotic balance, causing cell injury [24,25].

Cataract formation is also associated with hydrogen peroxide production via glucose auto-oxidation [27]. Aldose reductase, the key enzyme in the polyol pathway, catalyzes the conversion of glucose to the sugar alcohol sorbitol, which is ultimately converted to fructose by sorbitol dehydrogenase. As an osmolyte, sorbitol causes osmotic swelling, changes in membrane permeability, glutathione loss, myo-inositol loss, free radical formation, and hydrogen peroxide, all of which contribute to diabetes complications as cataracts, retinopathy, and neuropathy [28,29]. Higher concentrations of hydrogen peroxide cause tissue damage and clouding of the lens.

Special glasses, anti-glare glasses, or magnifying lenses can help with the early symptoms of cataracts, and if they are not treated, surgery is the treatment of choice for cataracts [30,31,32,33]. However, cataract surgery is costly with several consequences: endophthalmitis, posterior capsule rupture, postoperative macular edema, and posterior capsule opacification [34]. Besides that, cataract surgery changes the shape of the corneas and this treatment occasionally causes presbyopia. Presbyopia is the physiological degradation of accommodation or loss of accommodation power due to nuclear cataract. One of the disadvantages of cataract surgery is a lack of true accommodative ability. The loss of accommodative power is essentially due to the progressive failure of the capsule to mold the lens into a more spherical shape [35]. Therefore, searching for safe substances that can reduce the risk or delay the onset of cataracts is an essential step in developing cataract treatments.

### 2.2. Free Radicals Contribute to Cataract Formation 

The electrons of an atom are arranged into orbitals, each of which can accommodate a different pair of electrons. Free radicals are molecules that have only one electron in their outermost orbital or an unpaired electron [36]. Free radicals will take electrons from each adjacent molecule to be stable, triggering cell damage. When each molecule gains or loses electrons, free radicals are produced. Free radicals can be created in the body in two ways: physiologically as part of normal metabolic processes, or pathologically due to illness [37,38,39].

The primary physiological source of free radicals is cellular respiration [40]. An electron transport chain carries electrons from complex to complex and ultimately to oxygen, providing a proton gradient that is utilized to make ATP. The process of generating ATP by donating electrons to the complex in the inner mitochondrial membrane is known as oxidative phosphorylation. In the last part of this process, a cytochrome c oxidase molecule transforms electrons into oxygen [40].

**Figure 3:**
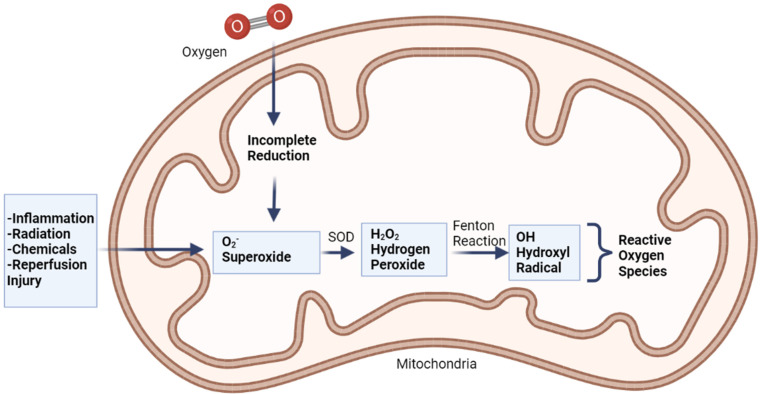
Production of free radicals via Fenton reaction, adapted from Coleman (2010) [42].

When oxygen accepts four electrons, it usually turns into water. If oxygen does not take all four electrons, it will have an unpaired electron in its orbital, which will cause free radicals to develop. Superoxide is produced when oxygen is supplied with only one electron (O_2_). It produces hydrogen peroxide (H_2_O_2_) with two electrons and hydroxyl radical with three electrons (Figure 3) [41].

Free radicals can also be produced as a result of a pathogen. First, during inflammation, phagocytes such as macrophages can produce free radicals. Phagolysosomes are formed when infections enter the body and are consumed by phagocytes. NADPH oxidase, triggered by lysosomal enzymes and causing NADPH to be oxidized, losing two electrons, is also present in these phagocytes. These electrons can be captured by nearby oxygen molecules, forming O_2_ ions [43]. Superoxide dismutase (SOD), another enzyme, may combine these ions with hydrogen ions to create hydrogen peroxide. A respiratory burst (also known as an oxidative burst) is a process that results in the production of superoxide ions and hydrogen peroxide. Furthermore, phagocytes include a kind of nitric oxide synthase (eNOS), an enzyme that produces nitric oxide, which aids in the killing of infections [44]. On the other hand, nitric oxide reacts with superoxide ions to produce peroxynitrite free radicals (ONOO^−^). These ions and chemicals kill bacteria by rupturing cell membranes and disrupting protein synthesis [45].

**Figure 4:**
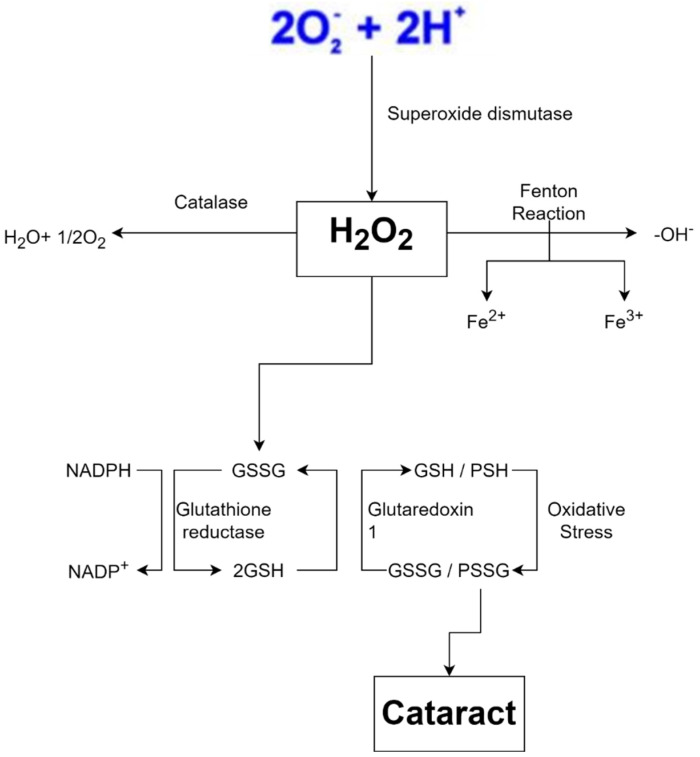
Oxidative stress is a key feature of cataract formation [47,48,49].

Free radicals are also produced by exposure to ionizing radiation such as X-rays. Radiation steals electrons from water in tissues, converting them into hydroxyl radicals. When metals such as copper or iron accumulate in the body, free radicals are produced. Hemochromatosis, for example, is a condition in which the body absorbs too much iron. Excess iron is oxidized by hydrogen peroxide, yielding iron 3^+^, hydroxyl radicals, and hydroxide ions as byproducts; iron 3^+^ may then be reduced to iron 2^+^ by hydrogen peroxide, yielding peroxide radicals and protons, and the cycle can be repeated indefinitely. As a result, the Fenton reaction can break down H_2_O_2_ to OH^-^ in the presence of transmission metals, such as Fe^2+^ or Cu^2 +^. Fenton reaction also produces free radicals, including numerous ROS such as superoxide anion radical (^•^O_2_^−^), H_2_O_2_, and hydroxyl free radical (^•^OH), and may lead to structural damage of the crystalline lens and contribute to cataract formation (Figure 4) [4]. This harms cells in numerous organs over time, resulting in cell death and tissue fibrosis [46]. 

Ischemia, or lack of blood flow to organs or tissues, also generates free radicals. Ischemic damage can cause mitochondria to produce ROS. Reperfusion occurs when blood flows back into is chemical tissue, carrying extra oxygen. When all this oxygen combines with pre-existing free radicals, it causes more cellular damage. Ischemia-reperfusion injury (IRI) is the medical term for this [50]. Free radicals are also produced when chemicals or drugs enter the body and are metabolized by the liver. Many free radicals are created when the liver metabolizes medicines such as acetaminophen or paracetamol (the primary active ingredient in TYLENOL^®^ products), which may cause considerable liver damage [51].

Because the body creates free radicals under normal circumstances, defensive systems are in place to keep them in check. Antioxidants such as vitamin A, C, and E, for example, deliver electrons that neutralize free radicals and protect cells [52]. Glutathione, another chemical in our body, functions as an antioxidant and neutralizes H_2_O_2_. To work properly, the two glutathione must be in a reduced form, allowing them to donate electrons and protons to H_2_O_2_ and turn it into harmless water. Because this mechanism oxidizes glutathione, glutathione reductase needs reduced nicotinamide adenine dinucleotide phosphate (NADPH) as an electron donor to restore glutathione to its functional state before restarting its activity. NADPH forms nicotinamide adenine dinucleotide phosphate (NADP^+^) after losing electrons. To replenish the supply of NADPH, an enzyme called glucose-6-phosphate dehydrogenase (G6PD) oxidizes glucose-6-phosphate and converts NADP+ to NADPH. Since glucose-6-phosphate is a byproduct of glucose, humans usually have large amounts of this substance as long as they are not starving [53].

Metal-carrying proteins, which attach to metal ions and assist in transporting or storing them, are another protective mechanism. This mechanism fights free radicals as if the ions were hidden so they could not form free radicals. Transferrin, which binds to iron, and ceruloplasmin, which binds to copper, are two examples of proteins attaching to metals and transporting them through the bloodstream. On the other hand, free radical scavenging enzymes transform free radicals into non-toxic molecules such as water. The enzyme SOD converts superoxide to hydrogen peroxide. In peroxisomes, catalase (CAT) converts hydrogen peroxide to water, while glutathione peroxidase in the cytoplasm does the same. When the amount of free radicals created surpasses this defensive system, cell damage ensues [54,55].

### 2.3. Natural Ingredients’ Potential as an Alternative Cataract Treatment

There have been attempts to employ herbal medicines to prevent cataract advancement based on the model of cataract development and the mechanism of its production route. Natural antioxidant molecules have been reported to have an inevitable application in cataract prevention and control due to their easy availability and fewer complications [56]. These biomolecules are excellent at preventing other molecules from oxidizing and producing free radicals. These free radicals set off a chain reaction, causing all lens cells to be damaged. Most of these antioxidants are reducing agents, such as thiols or polyphenols, which inhibit free radical chain reactions. Flavonoids, phenolic acids, carotenoids, vitamins, and lactoferrin are natural antioxidant compounds with anticataract action [57].

In fact, many antioxidants derived from plants such as curcumin, vitamin C, and vitamin E have been well recognized as potential anticataractogenic therapeutics. Vitamin C has been shown to be effective against UV-induced cataracts and age-related cataracts. It also prevents nuclear cataract. Vitamin C also scavenges free radicals. Vitamin E has been shown to be effective against both UV-induced and age-related cataracts by postponing galactose and amino thiazole-induced cataract, inhibiting lipid peroxidation, and maintaining membrane integrity. Curcumin was discovered to be an effective free radical scavenger due to its cytoprotective effect on glutathione-S-transferase enzymes and its efficacy against hyperglycemia, galactose, and naphthalene-induced cataract. Curcumin can also inhibit NFκB [12]. This section provides an overview of the various categories of plant-derived compounds that have been evaluated for potential as anti-cataracts.

### 2.4. Antioxidant Activities of Plants in Preventing Cataractogenesis

#### 2.4.1. Antioxidant Activities of Plants

The function of oxidative stress in cataract formation has been well documented [58,59]. As a result, antioxidants and free radical scavengers might be used as therapeutic techniques to treat cataracts. The study of antioxidants is growing because of their protective role in food and pharmaceutical products against oxidative damage in the body and pathological processes mediated by oxidative stress [60]. To obtain good antioxidant activity, several things need to be considered, such as using the type of solvent, as [61] reported. The antioxidant activity of the methanol extract of *Torilis leptophylla* L. crude and its derivative fractions was found to be varied. In addition, screening plant antioxidant properties and their derivative compounds require appropriate methods [60]. Therefore, this review examines previous studies related to antioxidant activity derived from plants.

This difference in antioxidant activity appears from the difference in the degree of polarity between the solvents used. The results of one-way analysis of variance (ANOVA) obtained in [62] showed that the extraction yield, phytochemical content, and antioxidant properties were significantly affected (*p* < 0.05) by the polarity of the extraction solvent. The results of other studies related to the different types of solvents on antioxidant activity were carried out by [63], who extracted *Sargassum serratifolium* leaves using various solvents such as ethyl acetate, ethanol, methanol, acetone, n-hexane, chloroform, and water. According to the study’s findings, ethanol is the most efficient extraction solvent and has the potential to operate as a natural antioxidant. Extraction in highly polar solvents yields high extracts but low phenolic and flavonoid content compared to non-polar ones [62]. The increase in total antioxidant activity and polarity-dependent reducing properties indicated the extraction of strong antioxidant compounds in polar solvents.

In addition to being influenced by the solvent used, antioxidant activity in several works of literature is also related to total phenolic and flavonoid levels. Research conducted by [64] has shown a strong association between antioxidant activity and total flavonoid content of many varieties of Nepalese vegetables.

Plant secondary metabolites with an aromatic ring containing at least one hydroxyl group are phenolic compounds and natural flavonoids [65]. Because their hydroxyl groups can directly contribute to antioxidant activity, phenolic substances are effective electron donors [66]. In addition, several of them promote the production of endogenous antioxidant molecules in cells [67]. According to various studies, free radical inhibition, peroxide decomposition, and metal inactivation are all properties of phenolic compounds [68]. The research conducted by [69] has shown a correlation between total phenolic content with total antioxidant capacity and lipid peroxidation inhibitory activity in in vitro studies. 

Previous reports showed that *Sargassum serratifolium* extracted using several solvents exhibited different total phenolics and antioxidant activities [63]. In addition to differences in solvent types related to polarity, plant preparation methods were also reported to affect antioxidant activity, such as research on fresh leaves and dried leaves of *Datura metel* L., (Amethyst) plants extracted with several solvents. The tendency of the content is the same, but the antioxidant activity test shows a difference where the antioxidant activity of dry crude extract equivalent to DPPH is on the order of butanol > chloroform > ethyl acetate extract > methanol > hexane extract. However, the order of antioxidant activity of the fresh organic crude extract against DPPH was methanol > hexane > chloroform > ethyl acetate extract > butanol [70]. Table 1 below shows some of the plants reported to have antioxidant activity.

Table 1 shows that the strength of antioxidant activity in plants is affected by several factors such as polarity of the solvent extraction, growth location plant species, and mode of action of antioxidant compounds present in a sample. These factors need to be studied more deeply to understand the potency of plant species to obtain maximum antioxidant activity. The white horehound shows relatively high antioxidant activity. The highest EC_50_ is the MVA extract of *Marrubium vulgare* L. leaves, with an EC_50_ of 6.43 ± 0.0411 mg/mL. Ginger also showed promising results. The highest IC_50_ is the methanol extract of Nigerian *Zingiber officinale* with a FRAP assay result of 89.15 ± 0.29 μg/mL. Antioxidant activities of ginger extracts were also studied in acetone extract, which has a maximum IC_50_ value of 0.654 and 0.812 mg/mL.

#### 2.4.2. Cataract Treatment with Herbal Plants

A cataract is a complex illness with several risk factors. Oxidative stress is a key factor in the onset and progression of cataracts [84,85]. An assessment of the contribution of this mechanism to cataract formation was carried out in a model of induced cataracts in experimental animals. A selenite-induced cataract is one of the good models of senile nuclear cataract and is very rapidly induced [86]. Degradation of calcium homeostasis increased ROS or free radical generation, calpain (calcium-activated protease) activation, insoluble protein, crystal precipitation, phase change, and cytoskeletal loss are the major causes of selenite-induced cataracts [87]. The eye lens possesses a robust antioxidant system as a defensive mechanism against harmful damage from ROS or free radicals. This system contains antioxidants such as reduced glutathione and antioxidant enzymes such as SOD, CAT, and glutathione reductase/peroxidase (GR/Gpx) [88]. 

Free radicals can cause gene mutations that lead to the formation of cataracts. Free radicals compete with electrons from intracellular molecules resulting in lipid peroxidation, protein modification, lesions on chromosomes, and mitochondrial DNA. This can result in impaired transmission and gene expression and react with DNA chains that also cause mitochondrial DNA (mtDNA or mDNA) damage. This DNA damage disrupts the gene regulatory system, interfering with protein regulation and expression. Mutations in the R48C gene impair A-crystallin stability, associated with lens opacities [89,90]. 

Meanwhile, free radicals also can cause autophagy, necrosis, and apoptosis of tissues. The regulation of the autophagic system in the body depends on the autophagic flux process, which is responsible for removing abnormal proteins. This results in impaired autophagosome binding to lysosomes, resulting in the accumulation of p62 (a classical receptor of autophagy). This accumulation activates caspases which then increase apoptosis due to the activation of factor-kappa B (NF-κB) [90,91]. 

**Figure 5:**
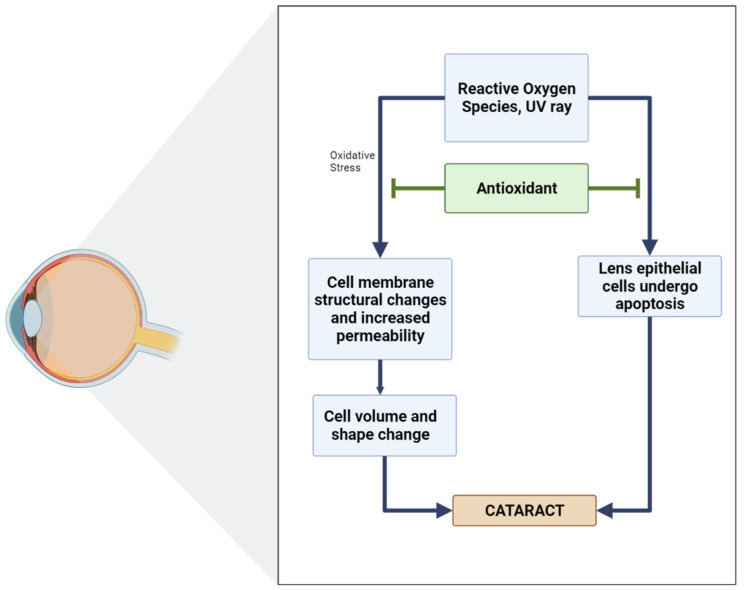
Diagram of the role of antioxidants in inhibiting cataracts [96].

In biological systems, the balance between oxidants and antioxidants is of the utmost importance, which has both physiological relevance (beneficial) and pathological consequences (which usually lead to the formation of diseases, for example, cataracts). Several studies have shown a positive relationship between antioxidant intake and a reduction in the incidence or development of cataracts (Figure 5) [92]. 

In animal experiments with this condition, compounds of plant origin and herbal medicine have also been demonstrated to have anticataract potential. Quercetin, a flavonoid found in fruits and vegetables, is a potent antioxidant and free radical scavenger with various health advantages, including cardioprotective, anti-diabetic, anti-inflammatory, and anticancer properties [93]. In the study [94], in Sprague Dawley mice, quercetin reduced the onset and development of selenite-induced cataracts and maintained lens chaperone function. In another study, intraperitoneal injections of citrus flavonoids prevented selenite-induced lenticular opacities in Wistar rats, with a corresponding increase in antioxidant enzyme activity, CAT, SOD, glutathione peroxidase (GSH-Px), glutathione S-transferase (GST), and glutathione reductase (GSH-Rx), as well as a reduction in lipid peroxidation, when compared to lenses treated only with selenite [95].

Curcumin is a brilliant yellow chemical with antioxidant qualities that is derived from the Zingiberaceae family’s Curcuma longa plant. Curcumin inhibits the formation of cataracts produced by galactose, oxidative stress, and streptozotocin by inhibiting lenticular antioxidants, lipid peroxidation, and the maintenance of soluble protein content. In Sprague Dawley mice, Nakazawa et al. (2017) found that both oil-soluble antioxidant compounds and water-soluble antioxidants may prevent the onset and progression of selenite-induced cataracts while still maintaining lens chaperone activity [97,98,99].

The report [100] stated that the ethanolic extract of the leaves and stems of *Cineraria maritima* showed promising results in treating cataracts in the eye lens of goats. According to the ethanol extract of the leaves of the binahong plant, the lens group of the goat lens induced with glucose and the addition of the binahong (Anredera cordifolia (Tenore) Steenis) extract exhibited more transparent results than the lens group induced with 55 mM glucose concentration. Binahong can suppress malondialdehyde generation at doses of 100 or 200 μg/mL [101]. In another study, it was stated that Lupeol, a pentacyclic triterpenoid isolated from *Vernonia cinereal*, was effective in the treatment of cataracts in the eye lens of Sprague Dawley rats induced by selenite from the results of testing biochemical parameters such as activity of SOD, CAT, GPx, GR, GST, Ca^2+^ ATPase, glutathione, ROS, and lipid peroxidation product (malondialdehyde) were found to be effective in the treatment of cataracts with lupeol [100,102].

Another study found that the root extract had more antioxidant activity than the leaf extract of the two extracts tested. This conclusion was corroborated by the presence of more apparent antioxidant components in the ethanolic extract of *L. aspera* root. The root extract of aspera root was tested in the lenses of cultured Wistar rats for probable anticataractogenic potential. The results showed that when the extract was combined with the extract aspera root ethanol in the lenses of selenite-induced Wistar rats, mean enzymatic antioxidant activity, mean levels of reduced glutathione, and mean malondialdehyde expression levels of genes encoding A- and B1-crystalline proteins were kept close to normal, and mean levels of crystalline proteins themselves were kept close to normal [103]. Kaemoferol, for example, is a natural flavonol, a secondary metabolite found in many plants, reveals effectiveness for anti-inflammatory and antioxidant properties. This compound also demonstrated therapeutic antiglaucoma efficacy through suppressing ocular hypertension, inflammation, and oxidative stress [104]. Table 2 shows the results of the analysis of several types of plants that are reported to be able to be used in cataracts management.

Based on several references, as shown in Table 2, it can be seen that the use of plant extracts shows promising results in overcoming the problem of cataracts. The induction cataract model can show the effectiveness of the extracts given. In addition to plant extracts, nanoparticles synthesized from plants have also demonstrated effectiveness in treating cataracts, as reported by [109], where the nanoparticles synthesized from shallots showed good anticataract activity compared to shallot extracts that were not synthesized into nanoparticles. Another study investigated the antioxidant capacity and efficiency of silver nanoparticles (AgNPs) biosynthesized using an ethanolic extract of *Tabernaemontana divaricata* leaf in preventing selenite-induced opacification of the ocular lens in vitro (cataractogenesis). The activity of CAT, SOD, GPx, and GST, as well as levels of reduced glutathione and malondialdehyde, were measured in this investigation. The ethanolic extract of *T. divaricata* and AgNPs biosynthesized using *T. divaricata* extracts exhibit excellent in vitro antioxidant activity and the capacity to inhibit experimental selenite-induced opacification in Wistar mice’s lenses, according to the findings [114].

Several in-vivo studies have also proved the ability of plant products to have a positive effect on cataract [11]. Streptozotocin (STZ)-induced diabetic rats were used in the in vivo experiment by Chung et al. At 11 weeks following STZ injection, diabetic control rats acquired cataracts, but oral *Aralia elata* extract provided at 300 and 600 mg/kg body weight for 11 weeks decreased cataract formation by 15% and 12%, respectively [115]. 

The research looked at whether highbush blueberry leaf polyphenols could help prevent cataracts and the reasons behind it. HPLC-DAD was used to measure chlorogenic acid, quercetin, rutin, isoquercetin, and hyperoside in *Vaccinium corymbosum* leaf decoction (BBL). On postnatal days 11 and 12, Wistar rats were administered subcutaneously with 20 μmol selenite (Na_2_SeO_3_)/kg body weight or intraperitoneally with 100 mg dry BBL/kg body weight. Only normal saline was given to the control group. BBL considerably reduced lens opacification, according to a cataract examination. It also protected the lens from oxidative selenite assault, calpain activation, and protein loss and aggregation [116]. In model rats, rosmarinic acid, a polyphenol found in rosemary *(Rosmarinus officinalis*), was confirmed to delay cataract development and lower the degree of lens opacification [116].

Natural substances containing antioxidants or secondary anti-inflammatory metabolites may serve as anticataract agents in modern herbal medicine, which has played a significant role in treating oxidative stress and its consequences [117]. In most instances, free radicals cause lens opacity [12], and protein alteration by free radicals is also a result of extreme oxidative stress. Some plant-based substances can inhibit protein insolubilization, delaying lens opacification [12]. Natural chemicals that are antioxidants or secondary anti-inflammatory metabolites have the potential to be the most effective anticataract treatments. Antioxidant effects are one of the primary mechanisms for cataract prevention in most instances. However, not all plants with antioxidant potential can have anticataract properties. Plant polyphenols have been known to have an anticataractogenic effect has been thoroughly investigated in vitro and in animals [118,119].

As reported in the literature, the chemical structure of many antioxidants plays an important role in preventing ocular disease progression. The effect of aromatic ring number in phenolic compound-conjugated chitosan injectables was investigated with the purpose of developing a more sophisticated drug carrier with significant anti-inflammatory and antioxidant characteristics. Low and high numbers of aromatic rings might have negative effects on injectables’ pharmaceutical uses; however, a molecule with a moderate ring number has been shown to be the most effective agent for improving drug delivery and giving chitosan injectables medicinal qualities. The intracameral infusion of kaempferol-conjugated pilocarpine, which can treat progressive glaucoma by concurrently exerting various pharmacological actions to decrease ocular hypertension, inflammation, and oxidative stress, shows extraordinary efficacy [104].

### 2.5. Other Natural Ingredients Besides Antioxidants That Can Inhibit Cataracts

#### 2.5.1. Natural Antioxidant as Antiglycation Agent

Glycation is a phenomenon which is caused by increased glucose level in skin fibers. Glycation, also known as Maillard reaction, is a non-enzymatic reaction adduct formation between amino groups and carbonyl compounds. Glycation process occurs through oxidation, dehydration and cyclization reactions, and irreversible compounds, called advanced glycation end products (AGEs). During healthy aging, AGEs are formed at accelerated rates in diabetes, and also as causative factors for pathogenesis of diabetes, neurodegenerative disease, and cataracts [120].

Protein glycation changes the biological activity of proteins and starts the breakdown process; therefore, stopping it can help people with diabetes avoid significant consequences. With aging, advanced glycation end products (AGEs) build up in the lens, causing opacities [121]. Non-enzymatic interactions between the amino groups of proteins and the carbonyl-reducing sugars create the primary problems of diabetes (one of which is cataracts). Attempts to impact protein glycation have been made in a variety of ways. Various natural and synthetic substances, including flavonoids, phenol derivatives, imidazoles, Schiff bases, thiazolidines, and sulfates, have been shown to suppress protein glycation and the formation of AGE products. There are several mechanisms involved, including capturing reactive amino groups and preventing them from reacting with glucose or capturing carbonyl compounds, chelation with glycation-catalyzing trace metal ions, radical scavenging, and inhibition of oxidative degradation of metal catalysts for glucose or various glycated protein intermediates. By avoiding AGEs buildup, antiglycation treatment will become a feasible approach for managing advanced diabetic complications [122].

**Figure 6:**
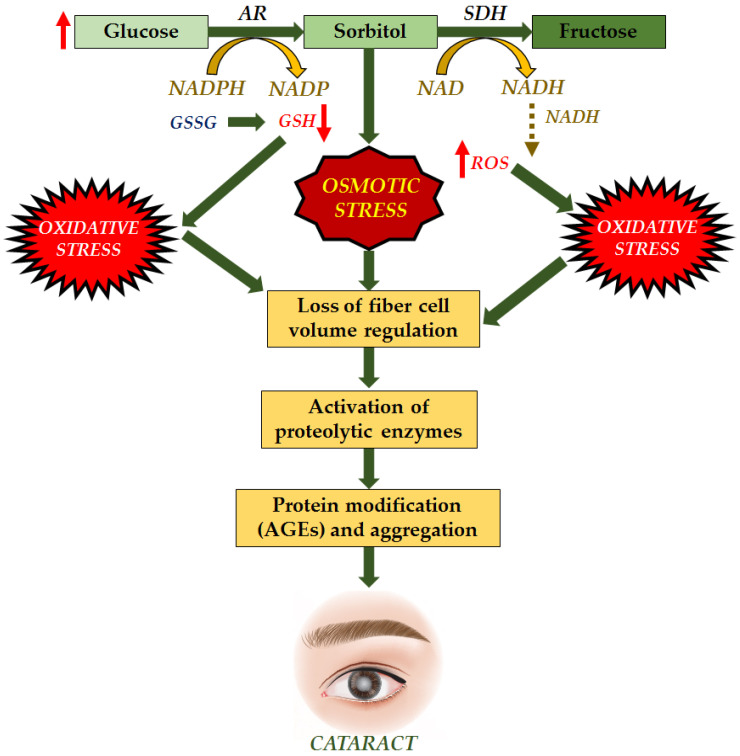
Molecular mechanisms of AGEs for formation of cataract. Increase in glucose led to decrease in glutathione and increase in ROS induced osmotic stress and oxidative stress, synergistically inhibiting the ability of fiber cell results in the activation of enzymes and leading to formation of AGEs and formation of cataract [125].

Other natural substances such as quinic acid from *Erigeron annuus* was reported to exhibit the most potent inhibitory activity against AGE formation and prevented opacification of rat lenses. This compound also has been reported to act as an inhibitor of RLAR (rat lens aldose reductase), AGE formation, AGEs–BSA cross-linking, and cataractogenesis. The molecular mechanisms of AGEs in the formation of cataracts are presented in Figure 6 [123,124]. 

#### 2.5.2. Natural Antioxidant of Plant as Aldose Reductase Inhibitors in Cataractogenesis

In diabetes, chronically elevated blood glucose plays a crucial role in determining complications such as cataracts. Aldose reductase converts glucose to sorbitol during hyperglycemia, while sorbitol dehydrogenase catalyzes the conversion of sorbitol to fructose via sorbitol dehydrogenase. Because the polyol pathway is involved in the etiology of diabetic cataracts and AR is the rate-limiting enzyme of the polyol pathway, sorbitol cannot cross cell membranes, causing cell swelling, degeneration, and necrosis. Therefore, it has been hypothesized that AR inhibition could be a pharmaceutical target in managing diabetic cataracts. AR has a role in various disease pathological processes by regulating cytokines, growth factors, oxidative stress, and other intracellular signal transduction pathways. The binding site of the AR inhibitor is a large hydrophobic pocket that serves as the target [126]. As a result of the polar and non-polar interactions between the inhibitor and the complementary residue corresponding to the enzyme-binding pocket, binding of the inhibitor occurs. The selectivity of the inhibitor is thought to be mainly due to the interaction of the inhibitor enzyme in the non-polar domain [127,128].

**Figure 7:**
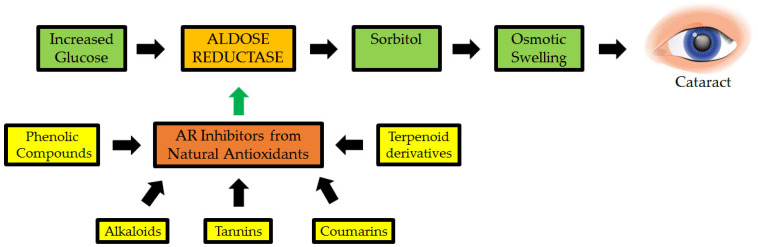
Natural antioxidants as possible inhibitors of aldose reductase (AR: a key enzyme implicated in cataractogenesis) [130].

Polyphenols in *Eleusine coracana* are an important anti-diabetic and natural antioxidant component. They were tested for their ability to suppress AR in a study of cataractogenesis (Figure 7). Syringic, ferulic, trans-cinnamic acids, p-hydroxy benzoic, p-coumaric, gallic, protocatechuic, vanillic, and quercetin, among other phenolic elements in *Eleusine coracana*, significantly suppressed cataract eye lens, with the latter being more active, with an IC_50_ of 14.8 nM. Polyphenols present in the seed coats of *Eleusine coracana* plants have been reported to suppress AR in a reversible, non-competitive manner. As a result, the findings add to the body of evidence supporting *Eleusine coracana* ability to suppress cataractogenesis in people [129].

In Chinese traditional medicine, *Chrysanthemum indicum* L. blooms are used to treat eye diseases. On rat lens AR, the inhibitory activity of components extracted from this plant’s active fractions was investigated. Luteolin, acacetin-7-O-(600-a-L-rhamopyranosyl)-b-D-glucopyranoside, and chlorogenic acid were found to be effective inhibitors [11]. Isolated from the methanolic extract of the dried leaves of *Manilkara indica*, the C-glucosyl flavone, isoaffinetin, inhibited AR in bovine lens, rat lens, and human recombinant. Isoaffinetin, like many other flavonoids, works by inhibiting *dl*-glyceraldehyde and NADPH in a noncompetitive manner. The quantity of hydroxyl groups in ring B increases C-glucosyl flavone inhibition, according to a structure–activity connection study [131].

Another study created curcumin analogues and tested their potential to block the enzyme. Curcumin analogues with ortho-dihydroxyl groups create a tighter binding to AR, allowing them to display strong action, according to structure–activity relationship studies [132]. The OH group at position 4 was found to be crucial for AR inhibitory property in a structure–activity connection investigation. AR action is also inhibited by the presence of an O-methyl group close to the carbon bearing the phenolic OH moiety. The noncompetitive inhibition of AR by phenolic acids was discovered to be reversible [129].

#### 2.5.3. The Potential of Natural Antioxidant as Antiapoptotic against Cataractogenesis

Apoptosis by ocular lens epithelial cells also contributes significantly to cataract progression. There are many mechanisms of cataracts that ultimately lead to lens cell apoptosis and impair vision. For this reason, one of the benefits of natural compounds in plants against cataracts is the inhibition of the epithelial cells of the eye lens to perform apoptosis. Many pathways involved in apoptosis are classified as intrinsic and extrinsic pathways, depending on different apoptotic triggers. Lens opacity is related to mitochondria-dependent processes. Radiation, drugs, toxins, and free radicals cause mitochondrial damage and malfunction. These lead to the release of pro-apoptotic proteins (such as cytochrome c and second mitochondrial activator of caspases, SMAC) from the inner mitochondrial surface into the cytosol, resulting in programmed cell death. Oxidative stress in cataract formation has been identified as a critical mediator of apoptosis in lens epithelial cells [133,134].

Green tea’s most prevalent component, epigallocatechin gallate (EGCG), is a powerful antioxidant. In HLEB-3 cells, EGCG was found to protect against cell death caused by H_2_O_2_. H_2_O_2_-induced formation of ROS, loss of mitochondrial membrane potential (m), and cytochrome c release from the mitochondria into the cytoplasm were all reduced by EGCG. The H_2_O_2_-stimulated rise in caspase-9 and caspase-3 expression, as well as the drop in the Bcl-2/Bax ratio, were both suppressed by EGCG. Furthermore, EGCG prevented H_2_O_2_ from reducing the activation and expression of ERK, p38 MAPK, and Akt. These data imply that EGCG protects HLE cells against H_2_O_2_-induced mitochondrial apoptosis by modulating caspases, the Bcl-2 family, as well as the MAPK and Akt pathways [135].

Myricetin, a flavonoglycoside, is isolated from the stem, bark, branches, and fruits of Myrica rubra or other plant sources, with antioxidant properties have been known to act as a scavenger of ROS molecules. The antioxidant and anti-apoptotic role of myricetin has already been proven to decrease the level of ROS significantly. Myricetin can also inhibit the apoptosis of epithelial cells by increasing the levels of SOD, CAT, and glutathione through the Bax/Bcl-2 signaling pathway. Myricetin also inhibited the apoptosis of H_2_O_2_ stressed lens epithelial cells and through its anti-apoptotic potential, this compound is effective in preventing apoptosis-driven cataractogenesis of the human eye lens [136].

## 3. Conclusions

Currently, cataracts are still the leading cause of visual impairment. Cataracts are caused by a variety of factors, including tissue changes caused by aging in which proteins and lens fibers begin to break down, resulting in blurred or unclear vision, diabetes complications that cause high sugar levels in the aqueous humor, and oxidative stress caused by free radicals such as ROS. The way to neutralize ROS and other free radicals is with natural antioxidants. Antioxidants can donate electrons to make ROS and other free radicals less reactive. An online literature review revealed that many medicinal plants contain high antioxidant activity, such as amethyst leaves, passion fruit leaves, and ginger. Based on some literature that has been studied, it can be seen that many plants have bioactivity as anticataracts. Therefore, it is concluded that plants with high levels of antioxidants can be incorporated into cataract prevention efforts, and further research on cataract treatment can incorporate plants as a natural source of antioxidants that inhibit the progression of cataracts.

## Figures and Tables

**Table 1:** Summary of plants that have been reported to have antioxidant activities.

Plants and Parts Used	Solvent/Fraction		Content	Antioxidant Activity	Reference
*Torilis leptophylla* L.	Methanol (TLM)		Total phenolic content (TPC) (121.9 ± 3.1 mg GAE/g extract)	EC_50_ value (anti-radical) based on DPPH (41.0 ± 1 μg/mL), ABTS (10.0 ± 0.9 μg/mL), and phosphomolybdate (10.7 ± 2 μg/mL) tests for TLB, radical hydroxyl radicals (8.0 ± 1 g/mL) for TLC, superoxide radicals (57.0 ± 0.3 μg/mL) for TLM and hydrogen peroxide radicals (68.0 ± 2 μg/mL) for TLE were generally lower. Potential antioxidant properties.	[61]
	Fraction of n-hexane (TLH)		The total flavonoid content (TFC) of TLE (60.9 ± 2.2 mg RTE/g extract) was found to be significantly higher than the other solvent fractions.		
	Chloroform Fraction (TLC)				
	Ethyl acetate (TLE) fraction				
	Fraction of n-butanol (TLB)				
	Residual aqueous fraction (TLA)				
Fresh and dried leaves of *Datura metel* L. (Amethyst) Plant	Methanol	Fresh leaves	Alkaloids, flavonoids, saponins	The antioxidant activity of dry crude extract equivalent to DPPH. (2,2-diphenyl-1-picrylhydrazyl) was in the order of butanol > chloroform > ethyl acetate extract > methanol > hexane extract. However, the order of antioxidant activity of the fresh organic crude extract against DPPH (2,2-diphenyl-1-picrylhydrazyl) was methanol > hexane > chloroform > ethyl acetate extract > butanol.	[70]
		Dry leaves	Alkaloids, flavonoids, saponins		
	Chloroform	Fresh leaves	Alkaloids, saponins, tannins		
		Dry leaves	Alkaloids, saponins, tannins		
	Hexane	Fresh leaves	Saponins, tannins		
		Dry leaves	Saponins, tannins		
	Ethyl acetate	Fresh leaves	Alkaloids, saponins		
		Dry leaves	Alkaloids, saponins		
	Butanol	Fresh leaves	Alkaloids, flavonoids		
		Dry leaves	Alkaloids, flavonoids		
Nigerian *Zingiber officinale*	Methanol		The extract’s total phenol and flavonoid contents were 15.24 ± 0.02 mg GAE/g and 19.84 ± 0.32 mg/g CE.	DPPH test showed IC_50_ value 47.05 ± 2.03 μg/mL	[71]
				FRAP test showed IC_50_ value 89.15 ± 0.29 μg/mL	
The bark of *Phyllanthus Emblica* L.	Ethanol: water (7:3) (PEE)		Total phenol content 99.523 ± 1.91 (mg of GAE/g extract) Total Flavonoid Content 389.33 ± 1.25 (mg of quercetin hydrate/g extract) Total Tannin Content 310 ± 0.21 (mg of catechin/g extract)	Based on the hydrogen peroxide scavenging activity test, the ability to inhibit PEE (polyphenolic-enriched extract) free radicals depends on the PEE dose. At a 200 μg/mL concentration, the percentage of PEE inhibition (43.20%) was almost comparable to ascorbic acid (55.39%). However, at the concentration of PEE 250 μg/mL, the percentage inhibition of PEE was 79.62%, which was found to be better than ascorbic acid (71.34%). The IC_50_ PEE value was 188.80 μg/mL, while ascorbic acid was 177.7 μg/mL.	[72]
				Based on the ABTS ((2,2′-azino-bis(3-ethylbenzothiazoline-6-sulfonic acid)) assay, the free radical inhibitory activity of PEE was found to be concentration-dependent. The maximum inhibition of ABTS radicals at a 250 μg/mL concentration was 42.91%, which was less effective than the standard (ascorbic acid). The IC_50_ value of PEE was 329.20 μg/mL, while ascorbic acid was 133.96 μg/mL.	
*Isotome longiflora*	Ethanol		Flavonoids, saponins, triterpenoids, and alkaloids	IC_50_: value: 9.57 ppm	[73]
	n-hexane fraction		Steroids and alkaloids	IC_50_: value: 99.59 ppm	
	Chloroform fraction		Flavonoids, steroids, and alkaloids	IC_50_: value: 48.54 ppm	
*Sargassum serratifolium*	Ethyl acetate		TPC 105.0 ± 2.44 mg Phloroglucinol eq/g extract	Ethyl acetate, ethanol, and methanol extracts showed relatively strong DPPH, ABTs, and superoxide radical activities. The hexane and ethyl acetate extracts exhibited the most potent hydroxyl radicals and ROS scavenging activity. Sargahydroquinoic acid (SHQA), sargachromanol (SCM) and sargaquinoic acid (SQA) are the main antioxidant components in *S. serratifolium.*	[63]
	Methanol		TPC 100.9 ± 2.61 mg Phloroglucinol eq/g extract		
	Ethanol		TPC 100.2 ± 2.20 mg Phloroglucinol eq/g extract		
	Acetone		TPC 91.9 ± 0.65 mg Phloroglucinol eq/g extract		
	Hexane		TPC 53.7 ± 1.43 mg Phloroglucinol eq/g extract		
	Chloroform		TPC 53.2 ± 1.64 mg Phloroglucinol eq/g extract		
	Water		TPC 23.0 ± 1.57 mg Phloroglucinol eq/g extract		
*Arisaema jacquemontii* root Blume	Methanol		TPC 45 ± 1.7 GAE/g TFC (Total flavonoid compound) 35.5 ± 2.2 mg rutin equivalent/g	The extract had significant antioxidant activity in all assays, with 64.16 ± 0.19% in DPPH and 62.16 ± 0.17% in NBT (nitroblue tetrazolium) assays, and reduced Fe^3+^ ferricyanide complexes to form iron (Fe^2 +^).	[74]
Straw mushroom	Alcohol		The total phenolic content in the extract determined by the Folin–Ciocalteu method was 6.18 mg GAE/g extract	These results indicate that the ethanolic extract of *A. bisporus* has potent antioxidant activity and can be explored as a new natural antioxidant.	[75]
Passion Fruit (*Passiflora edulis*) Leaves	Aqueous		Total phenolic content 8.3 ± 0.22 mg GAE g	*P. edulis* leaf aqueous extract is a powerful source of antioxidants. The extract showed that it could reduce oxidative stress in vivo, increasing antioxidant power and lipid peroxidation in mice, especially in organs.	[76]
*Capparis spinosa*	Water:ethanol 20:80 (v/v)		Total phenol content 427.27 ± 3.21 (mg GAE/g dry matter) Flavonoids 57.93 ± 2.31 (mg QE/g dry matter) Anthocyanins 4.81 ± 0.85 (mg Cy-3-glu E/g dry matter)	DPPH test showed that plant extracts showed higher antioxidant activity than BHT (IC_50_ = 7.41 vs. 8.31 µg/ mL).	[77]
*Dendrobium sabin* flower (*DS*)	100% methanol (w/v), 100% ethanol (w/v), and 100% water (w/v).		100% methanol crude extract showed the highest total phenolic content (40.33 ± mg GAE/g extract)	The correlation between antioxidant activity and total phenolic content indicates that phenolic compounds are the dominant antioxidant components in this flower extract. Microbial fermentation on DS flower media showed the potential to increase the phenolic content and scavenging activity of DPPH.	[78]
Ginger	Ethanol, methanol, acetone, and ethyl acetate		The methanol extract showed the maximum phenolic content (1183.813 mg GAE/100 g in Ayikel and 1022.409 mg GAE/100 g in Mandura). The least phenolic content was found in acetone extract (748.865 mg GAE/100 g in Ayikel). and 690.152 mg GAE/100 g in Mandura)	The highest DPPH radical scavenging activity (84.868% in Ayikel and 82.883% in Mandura) was observed in methanol. However, acetone showed minor DPPH radical scavenging activity (73.864% in Ayikel and 70.597% in Mandura). The antioxidant activity of the ginger extract was also expressed as IC_50_ value_,_ and acetone extract had the maximum IC_50_ value (0.654 and 0.812 mg/mL), followed by ethyl acetate and ethanol, while methanol was the lowest (0.481 and 0.525 mg/mL).	[79]
*Chaptalia nutans* Daun leaves	Hydromethanol (30/70 methanol-water)		Quantitative studies of phytochemicals showed total phenols (30.17 ± 1.44 mg/g), flavonoids (21.64 ± 0.66 mg/g), and condensed tannins (9.58 ± 0.99 mg/g)	DPPH (345.41 ± 5.35 μg/mL) and FRAP (379.98 ± 39.25 μM FeSO4/mg sample).	[80]
Leaves of *Marrubium vulgare* L.	Hydroethanolic (MVE) and hydroacetonic (MVA)		The results showed that the total phenol content was higher in the MVA (112.09 ± 4.77 mg GAE/DW) than in the MVE extract (98.77 ± 1.68 mg GAE/DW). Total flavonoid content was also higher in MVA extract (21.08 ± 0.38 mg QE/g DW) compared to MVE (17.65 ± 0.73 mg QE/g DW).	Both extracts had good total antioxidant activity. DPPH and FRAP tests showed that MVE extract had better antioxidant activity, with IC_50_ = 52.04 μg/mL ± 0.2 and EC_50_ 4.51 ± 0.5 mg/mL, compared to MVA extract (IC_50_ = 60.57 ± 0.6 μg/mL and EC_50_ of 6.43 ± 0.0411 mg/mL).	[81]
Three species of bee propolis	Water extract		The highest TPC was found in the H. Fimbriata extract at 13.21 mg/mL, followed by the *T. Binghami* and *T. apicalis* extracts at 10.11 and 7.60 mg/mL, respectively. The highest TFC observed was from the aqueous extract of *H. Fimbriata* propolis, which was 34.53 mg/mL, while the lowest TFC recorded was from the extract of *T. binghami* species at 34.17 mg/mL. The aqueous extract of *T. apicalis* showed an average TFC value of 34.50 mg/mL	The results showed that the percentage of *H. fimbriata* DPPH scavenging activity (56.91%), especially at a concentration of 5 mL was higher than ascorbic acid (48.22%), *T. apicalis* (47.56%), and *T. binghami* (41.87%).	[82]
Tragopogon porrifolius	Water, 80% ethanol, and 100% ethanol			The results showed that the polarity of the extraction solvent affected TPC, TFC, and antioxidants.	[83]

**Table 2:** A list of plants and parts of plants used to prevent cataractogenesis.

Plants and Parts Used	Solvent	Test Animals	Results	Reference
Binahong (*Anredera cordifolia* (*Tenore) Steenis*)	Ethanol	Glucose-induced goat lens (ex vivo)	The lens group with added binahong extract had more transparent outcomes than the lens group induced with 55 mM glucose concentration). Binahong can suppress malondialdehyde generation at doses of 100 or 200.	[101]
Lupeol, a pentacyclic triterpenoid isolated from Vernonia cinerea	Ethyl acetate fraction of Vernonia cinerea methanol extract	Selenite-induced Sprague Dawley rat eye lens (in vivo)	Biochemical parameters such as the activity of SOD, CAT, GPx, GR, GST, Ca2+ ATPase, glutathione content, ROS, a lipid peroxidation product (malondialdehyde) was estimated and found to be effective in the treatment of cataracts with lupeol.	[102]
*Heliotropium indicum*	Water	10-day-old Sprague Dawley rat pups of both sexes (in vivo)	Cataract scores showed that the extract significantly reduced selenite-induced cataracts at all dose levels (*P* 0.001). Lens transparency markers (aquaporin 0, alpha A and B crystallins) and total lens protein and lens glutathione levels were significantly preserved (*P* 0.01–0.001). The extract exhibited relevant activities for free radical scavenging and lipid peroxidation inhibition. The integrity of the lens epithelium and fibers in histopathological assessment was maintained with *Heliotropium indicum* extract treatment.	[105]
*Foeniculum vulgare Mill*.	Petroleum ether, chloroform, and dichloromethane	Streptozotocin induced mice (in vivo)	Trans-anethole can effectively exhibit anticataract activity by increasing soluble lens protein, decreasing glutathione, CAT, and SOD activity on in vitro incubation of ocular lens with 55 mM glucose. Trans-anethole showing non-competitiveness for mixed type lens aldose reductase inhibition using Lineweaver–Burk plots.	[106]
*Cineraria maritime* leaves and stems	Ethanol	Goat eye lens (ex vivo)	From the DPPH (*2,2-diphenyl-1-picrylhydrazyl*) method, the IC_50_ value of the standard compound was found to be 5.45 μg/mL and that of the ethanolic extract of the plant was 73.26 μg/mL. The hydrogen peroxide method was the second method which was used for the determination of antioxidant potential. In this method, ascorbic acid was used as a standard which showed an IC_50_ value of 0.89 mg/mL, while the IC_50_ value of the ethanolic extract of the plant was found to be 1.30 mg/mL.	[100]
Chromolaena odorata leaves	*Ethanol extract Chromolaena odorata leaves* (ACO)	Streptozotocin-induced diabetic mice (in vivo)	ACO treatment resulted in substantial improvements in glucose and insulin tolerance, glycogen content, glucose absorption by skeletal muscle, serum insulin, and HDL-c levels, and a reduction in HOMA and lipid profile. Furthermore, by boosting endogenous antioxidants, ACO decreases oxidative stress. Moreover, ACO therapy significantly reduced the incidence and extent of cataracts.	[107]
Leaves of *Punica granatum*	Methanolic extract of Punica granatum leaves (MPGL)	Goat eye lens (ex vivo)	Reduced glutathione and SOD levels were lower in the cataract lens, indicating opacity. MPGL and quercetin treatment reduced opacity and increased antioxidant activity. *Punica granatum* leaves reduced glucose-induced cataractogenesis by inhibiting AR, reducing oxidative stress, and enhancing antioxidant defense mechanisms.	[108]
*Allium cepa (Onion)*	Extraction of flavonoids from onion peel and its combination with silver particles showed its activity as nanoparticles.	-	From the observations, the anticataract activity of silver nanoparticles from the *Allium cepa peel* showed better results than the *Allium cepa* *peel.*	[109]
Grape Seed Proanthocyanidin Extract (*GSPE*)	Proanthocyanidin	Selenite-induced cataract in mice (in vivo)	Administration of GSPE was able to maintain this antioxidant enzyme activity and anti-OH independently-ability, accompanied by a significant decrease in malondialdehyde, NO, Ca^2+^ and iNOS levels, and calpain-2 protein and mRNA expression.	[110]
Tephrosia purpurea	Water	Streptozotocin-induced rats (in vivo)	The results showed that the aqueous extract of *Tephrosia purpurea* prevented streptozotocin-induced metabolic disorders and cardiovascular complications and reduced the risk of cataract development.	[111]
Tephrosia purpurea	95% alcohol	Cataracts were induced by a single injection of sodium selenite (4 mg/kg, sc) into 9-day-old Sprague-Dawley rat pups (in vivo)	*T. purpurea* extract reduced core opacity in the lens while increasing insoluble protein, sulfhydryl protein, total nitrite, calcium levels, and Ca(^2+^)-ATPase activity. The extract reduces malondialdehyde levels while simultaneously preventing glutathione depletion.	[112]
*P. densiflora* pine bark	Extraction was performed using 60% EtOH in 50 °C for 3 h	Selenite-induced cataracts in the lens of Sprague Dawley rat pups (in vivo)	This study showed that the bark extract of *P. densiflora* independently could prevent cataract formation. Water-soluble protein, glutathione, SOD, glutathione peroxidase, and CAT activity levels were high. Conversely, water-insoluble protein, malondialdehyde and Ca^2+^-ATPase were low in the group treated with *P. densiflora* bark extract.	[113]
